# An Unusual Case of Gastrointestinal Bleeding

**DOI:** 10.1155/2011/748543

**Published:** 2012-01-22

**Authors:** Kristin N. Fiorino, Brian Lestini, Kim E. Nichols, Sudha A. Anupindi, Asim Maqbool

**Affiliations:** ^1^Division of Gastroenterology, Hepatology and Nutrition, The Children's Hospital of Philadelphia, Perelman, School of Medicine, University of Pennsylvania, Philadelphia, PA 19104, USA; ^2^Division of Oncology, The Children's Hospital of Philadelphia, Perelman School of Medicine, University of Pennsylvania, Philadelphia, PA 19104, USA; ^3^Division of Radiology, The Children's Hospital of Philadelphia, Perelman School of Medicine, University of Pennsylvania, Philadelphia, PA 19104, USA

## Abstract

A 10-year-old boy presented with a 3-day history of worsening abdominal pain, fever, emesis and melena. Abdominal ultrasound revealed a right upper quadrant mass that was confirmed by computed tomography angiogram (CTA), which showed an 8 cm well-defined retroperitoneal vascular mass. ^123^Iodine metaiodobenzylguanidine (^123^MIBG) scan indicated uptake only in the abdominal mass. Subsequent biopsy revealed a paraganglioma that was treated with chemotherapy. This case represents an unusual presentation of a paraganglioma associated with gastrointestinal (GI) bleeding and highlights the utility of CTA and ^123^MIBG in evaluation and treatment.

## 1. Introduction

Paragangliomas are rare tumors of neuroendocrine lineage with the potential for malignant progression. Among neuroendocrine tumors, paragangliomas may initially grow insidiously, lacking biochemical secretion [1], and therefore present with nonspecific symptoms dependent upon the site of origin. Abdominal paragangliomas typically present with signs of catecholamine hypersecretion and less often with tumor mass effects such as abdominal pain [2]. We report a case of vascular duodenal paraganglioma in a 10-year-old boy presenting with GI bleeding with the tumor invading the GI tract detected by CTA and ^123^MIBG imaging.

## 2. Case Report

A previously healthy 10-year-old African American boy presented with a 3-day history of fever and periumbilical abdominal pain and a 24-hour history of nonbloody, nonbilious emesis, tenesmus, and melena. Past medical and family histories were noncontributory. In the emergency room, he was dehydrated, afebrile, tachycardic, and normotensive. Abdominal exam was soft, with mild epigastric tenderness, without appreciable hepatosplenomegaly or masses. Stool was hemoccult positive. Initial laboratory studies were significant for hemoglobin 4 g/dL (13–16), MCV 80.1 fL (78–98), BUN 12 mg/dL (7–18), creatinine 0.8 mg/dL (0.3–0.8), and albumin 2.7 g/dL (3.7–5.6). Coagulation factors were normal but inflammatory markers were slightly elevated. Stool bacterial, viral, and parasitic pathogens were negative. Initial management included intravenous fluids rehydration followed by packed red blood cells. He was admitted to the GI service for further workup. Within 8 hours of presentation, abdominal examination suggested a new right upper quadrant fullness that quickly progressed from a doughy consistency to a discrete, dense 10 cm round mass in the middle of the abdomen. A broad differential diagnosis for GI bleeding and abdominal pain was entertained, including infectious etiologies, inflammatory bowel disease, Meckel's diverticulum, and malignancies. An extensive workup to investigate these possibilities followed.

**Figure 1:**
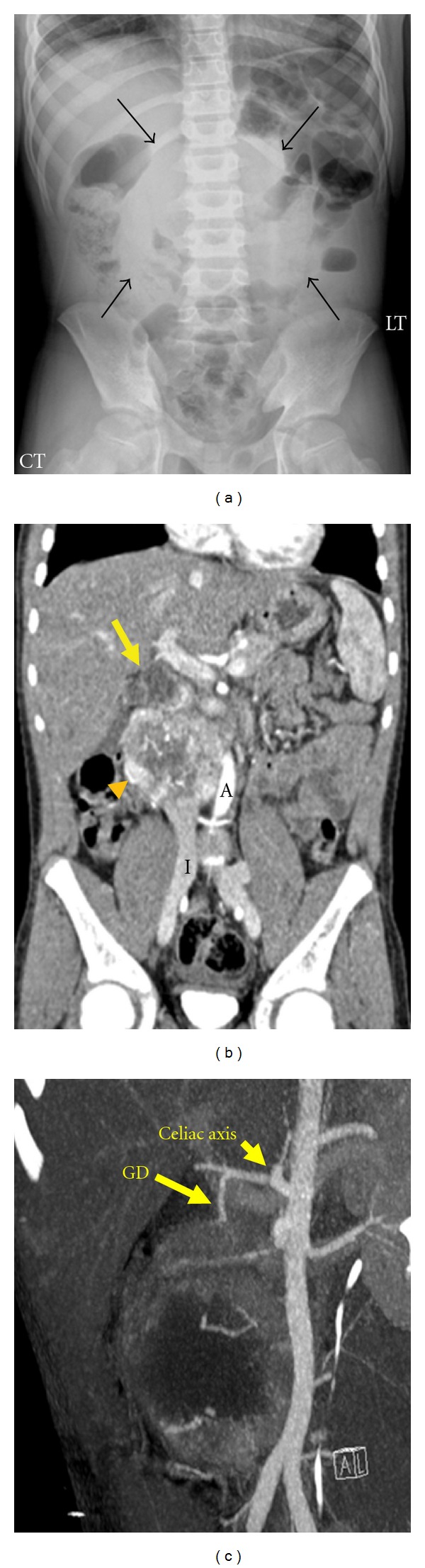
(a) Abdominal radiograph shows midline displacement of bowel loops by a soft tissue density (black arrows) without calcifications and no bowel obstruction. (b) Coronal CTA shows the markedly enhancing mass (orange arrowhead) displacing the aorta (A) and IVC (I) and its close proximity to the 3rd portion of the duodenum (yellow arrow). (c) A maximum intensity projection from the CTA depicts gastroduodenal artery GD (arrow) as the primary feeder of the paraganglioma.

Abdominal radiograph showed midline displacement of bowel loops. Abdominal ultrasound revealed a right upper quadrant mass displacing the pancreatic head and compressing the inferior vena cava (IVC). CTA indicated an 8 cm well-defined retroperitoneal mass. This highly vascular mass closely abutted the third portion of the duodenum with poor definition of the fat planes between mass and bowel, significantly compressing the IVC. The lesion was primarily fed by the gastroduodenal and superior mesenteric arteries. The mass also abutted the right kidney, resulting in pelvicaliectasis Figures 1(a)–1(c).

Given the concern for a GI tumor, staging evaluation including chest CT, brain magnetic resonance imaging (MRI), bone scan, and bone marrow biopsy were performed, all negative. ^123^MIBG scan indicated uptake only in the abdominal mass. Urinary catecholamines and tumor markers such as alpha-fetoprotein were negative. A CT-guided biopsy with histology and electron microscopy were consistent with a neuroendocrine tumor, specifically, a paraganglioma.

Over the course of 24 hours, he underwent exploratory laparotomy, which revealed a mass densely adherent to the third portion of the duodenum. Definitive upfront resection was deferred. Two cycles of neoadjuvant chemotherapy (vincristine, cyclophosphamide, and doxorubicin) resulted in no change in the mass size or degree of ^123^MIBG uptake. Surgical resection 5 months later revealed a tumor adherent to the inferior mesenteric vein, aorta, mesenteric vessels, vena cava, and ureter, which were dissected away. At the duodenal adhesion, a malignant ulcer was eroding into the third part of the duodenum. Near total gross resection with positive microscopic margins and negative lymph node sampling was achieved. Genetic testing revealed a germline point mutation in the mitochondrial complex II enzyme succinate dehydrogenase (*SDH*) gene, subunit B. 

## 3. Discussion

After the ultrasound and CT imaging, the differential was modified to include invasive GI tumors, including stromal tumors (GIST), neuroendocrine tumors, embryonal tumors, germ cell tumors, or tumors of nerve sheath origin. Rarely, tumors are associated with GI bleeding when eroding through intestinal mucosa [3].

Paragangliomas are exceedingly rare chromaffin tissue complexes of the neuroendocrine system, distributed along paravertebral and paraaortic axes. Paragangliomas commonly occur in the region between the inferior mesenteric artery and aortic bifurcation known as the organ of Zuckerkandl but can occur in all locations where paragangliomas are found [2, 4]. Paragangliomas can be either functional (sympathetic) or nonfunctional (parasympathetic) depending on the site of origin [2]. Paragangliomas are more frequent in adults, with few pediatric cases [5, 6]. Approximately 10–20% of pheochromocytomas and paragangliomas are diagnosed in childhood, with a slight predominance in males, particularly under the age of 10 [2]. Common presenting symptoms include GI bleeding, abdominal pain, and bilious emesis from obstruction [5]. Paragangliomas most often arise sporadically, with 10% arising in the setting of a hereditary predisposition [7]. Early age of onset, multifocal and bilateral tumors are more consistently associated with germline mutations, with rates of germline mutations in children younger than 10 years approaching 70% [7].

The specific gene defects associated with paraganglioma development involve the B, C, and D subunits of the SDH gene. While both B and D mutations increase the risk of malignant progression as compared to sporadic cases, SDHB-positive tumors are more aggressive [8–12], with nearly 70% eventually becoming metastatic [10]. Not only is the presence of an SDHB mutation considered to be a risk factor for development of metastatic disease, but it may also predispose to presentation of disease at an extra-adrenal location as well as at a younger age. Metastases have been identified in bones, liver, lungs, and lymph nodes. In a recent cohort study, the majority of patients had metastases to the bones, and all patients with SDHB mutations had at least one metastatic lesion to the bones [13]. Outcome in this group is typically poor [12]. Unbalanced chromosomal aberrations particularly in chromosome 11, 11p, and 1p are thought to be involved in childhood paragangliomas; aberrations in chromosome 11 are more common in malignant tumors [14]. Therefore, genetic testing of the index case and potentially affected family members is critical for management, surveillance, and genetic counseling. Patients and carriers of SDH mutations require long-term imaging surveillance of the neuroaxis, and routine surveillance for plasma catecholamine metabolites [8].

Radiographically, CT or MRI is the initial test of choice. Paragangliomas are vascular tumors that commonly contain necrotic, cystic, or hemorrhagic areas that can be identified by MRI. ^123^MIBG is a highly specific test that detects the catecholamine-secreting nature of a tumor. As ^123^MIBG testing is not 100% sensitive, other nuclear imaging modalities should be considered. [^18^F]FDG PET (positron emission tomography) is the preferred functional imaging modality for staging and treatment of SDHB-positive paraganglioma [15].

Treatment for localized paraganglioma is primarily surgical. For unresectable or metastatic paragangliomas, no clear standard of care exists. When indicated, sarcoma-like therapies are often employed. Combinations of cyclophosphamide, vincristine, and dacarbazine have been shown to induce tumor regression; however, the survival benefits of such therapy is unclear and has the potential for significant late effects in adolescent patients [16]. Experimental therapies employing high-dose ^131^MIBG (^131^iodine metaiodobenzylguanidine) are currently being evaluated for patients with metastases [17], and targeted small molecules that impact angiogenesis, such as sunitinib, may eventually prove to have utility in this disease [18].

Our patient had an SDHB-related paraganglioma. Parental testing revealed an identical mutation in the patient's mother, who has not experienced any tumors to date. Three of the patient's siblings have been tested and 2 carry germline SDHB mutations and are currently undergoing tumor surveillance. He has had follow-up imaging including MRI and ^123^MIBG exams every six months as well as annual head, neck, and chest imaging for metachronous tumors along the neuroendocrine axis. The patient was treated with 3 courses of ^131^MIBG therapy and is currently in remission on maintenance therapy with oral sunitinib. 

In summary, gastrointestinal bleeding from paraganglioma invading the duodenum is extremely rare in children. Although rare, neuroendocrine tumors should be entertained in the differential diagnosis of pain or GI bleeding, specifically if associated with an abdominal mass. Combinations of imaging techniques are key in the correct identification, characterization, evaluation, and management of these tumors.

## Abbreviations

CTA: Computed tomography angiogram
GI: Gastrointestinal
^123^MIBG: ^123^Iodine metaiodobenzylguanidine
^131^MIBG: ^131^Iodine metaiodobenzylguanidine
MRI: Magnetic resonance imaging
SDH: Succinate dehydrogenase.

## References

[1] Timmers HJLM, Pacak K, Huynh TT (2008). Biochemically silent abdominal paragangliomas in patients with mutations in the succinate dehydrogenase subunit B gene. *Journal of Clinical Endocrinology and Metabolism*.

[2] Waguespack SG, Rich T, Grubbs E (2010). A current review of the etiology, diagnosis, and treatment of pediatric pheochromocytoma and paraganglioma. *Journal of Clinical Endocrinology and Metabolism*.

[3] Rha SE, Byun JY, Jung SE, Chun HJ, Lee HG, Lee JM (2003). Neurogenic tumors in the abdomen: tumor types and imaging characteristics. *Radiographics*.

[4] Disick GIS, Palese MA (2007). Extra-adrenal pheochromocytoma: diagnosis and management. *Current Urology Reports*.

[5] Witkiewicz A, Galler A, Yeo CJ, Gross SD (2007). Gangliocytic paraganglioma: case report and review of the literature. *Journal of Gastrointestinal Surgery*.

[6] Atalabi OM, Lee EY (2008). Abdominal paraganglioma in a pediatric patient. *Pediatric Radiology*.

[7] Neumann HPH, Bausch B, McWhinney SR (2002). Germ-line mutations in nonsyndromic pheochromocytoma. *New England Journal of Medicine*.

[8] Benn DE, Gimenez-Roqueplo AP, Reilly JR (2006). Clinical presentation and penetrance of pheochromocytoma/paraganglioma syndromes. *Journal of Clinical Endocrinology and Metabolism*.

[9] Neumann HPH, Pawlu C, Pȩczkowska M (2004). Distinct clinical features of paraganglioma syndromes associated with SDHB and SDHD and gene mutations. *Journal of the American Medical Association*.

[10] Timmers HJLM, Kozupa A, Eisenhofer G (2007). Clinical presentations, biochemical phenotypes, and genotype-phenotype correlations in patients with succinate dehydrogenase subunit B-associated pheochromocytomas and paragangliomas. *Journal of Clinical Endocrinology and Metabolism*.

[11] Srirangalingam U, Walker L, Khoo B (2008). Clinical manifestations of familial paraganglioma and phaeochromocytomas in succinate dehydrogenase B (SDH-B) gene mutation carriers. *Clinical Endocrinology*.

[12] Amar L, Baudin E, Burnichon N (2007). Succinate dehydrogenase B gene mutations predict survival in patients with malignant pheochromocytomas or paragangliomas. *Journal of Clinical Endocrinology and Metabolism*.

[13] King KS, Prodanov T, Kantorovich V (2011). Metastatic pheochromocytoma/paraganglioma related to primary tumor development in childhood or adolescence: significant link to SDHB mutations. *Journal of Clinical Oncology*.

[14] Vicha A, Holzerova M, Krepelova A (2011). Molecular cytogenetic characterization in four pediatric pheochromocytomas and paragangliomas. *Pathology and Oncology Research*.

[15] Timmers HJLM, Kozupa A, Chen CC (2007). Superiority of fluorodeoxyglucose positron emission tomography to other functional imaging techniques in the evaluation of metastatic SDHB-associated pheochromocytoma and paraganglioma. *Journal of Clinical Oncology*.

[16] He J, Makey D, Fojo T (2009). Successful chemotherapy of hepatic metastases in a case of succinate dehydrogenase subunit B-related paraganglioma.. *Endocrine*.

[17] Gonias S, Goldsby R, Matthay KK (2009). Phase II study of high-dose [^131^I]metaiodobenzylguanidine therapy for patients with metastatic pheochromocytoma and paraganglioma. *Journal of Clinical Oncology*.

[18] Joshua AM, Ezzat S, Asa SL (2009). Rationale and evidence for sunitinib in the treatment of malignant paraganglioma/pheochromocytoma. *Journal of Clinical Endocrinology and Metabolism*.

